# Long-term course with iris changes after trabeculectomy for uveitic glaucoma associated with iris mammillation: a case report

**DOI:** 10.1186/s12886-023-02854-z

**Published:** 2023-03-15

**Authors:** Shinichi Usui, Tomoyuki Okazaki, Takahiro Fujino, Rumi Kawashima, Noriyasu Hashida, Kenji Matsushita, Eiichi Morii, Kohji Nishida

**Affiliations:** 1 Department of Ophthalmology, Osaka University Graduate School of Medicine, E7, 2-2 Yamadaoka, Suita, Osaka 565-0871 Japan; 2 Department of Pathology, Graduate School of Medicine and Faculty of Medicine, Osaka University, Osaka, Japan; 3 Integrated Frontier Research for Medical Science Division, Institute for Open and Transdisciplinary Research Initiatives, Osaka University, Osaka, Japan

**Keywords:** Iris nodule, Uveitic glaucoma, Iris mammillation

## Abstract

**Background:**

Iris mammillation is a rare disease characterized by the distribution of multiple nodules on the iris surface. The course of uveitic glaucoma with iris mammillation has never been reported.

**Case presentation:**

A 56-year-old woman, who presented with unilateral decreased vision, visited our hospital for treatment of uveitic glaucoma in the right eye. Multiple nodules were scattered over the iris surface in that eye. This case was diagnosed as iris mammillation on clinical findings. After excluding malignant tumors such as melanoma, trabeculectomy was performed. The resected iris had no pathologically malignant findings. The iris nodules evolved to a sand-like appearance, and the intraocular pressure remained stable without recurrent inflammation 7 years after trabeculectomy.

**Conclusions:**

In a case of unilateral uveitic glaucoma with iris mammillation, filtration surgery was performed after excluding the presence of a malignancy, and the long-term postoperative course has been stable.

## Background

Iris mammillation is a rare disease in which multiple nodules are distributed on the iris surface; the disease is generally unilateral and usually found on a dark iris or a nevus on the iris. Iris mammillation can be associated with ocular melanocytosis or phakomatosis pigmentovascularis type IIb and neurofibromatosis type I, which is usually sporadic but also may have an autosomal dominant inheritance pattern [1–6]. It has been suggested that iris mammillations may be an external sign of intraocular malignancy. In the case of associated melanocytosis of the iris, long-term follow-up is necessary because of the risk of uveal melanoma. Recently, an association between iris mammillation and prognosis of keratoconus has been reported, but its clinical significance has not been established [7]. Here, we report a case of glaucoma associated with inflammation, which has never been reported.

## Case presentation

**Fig. 1:**
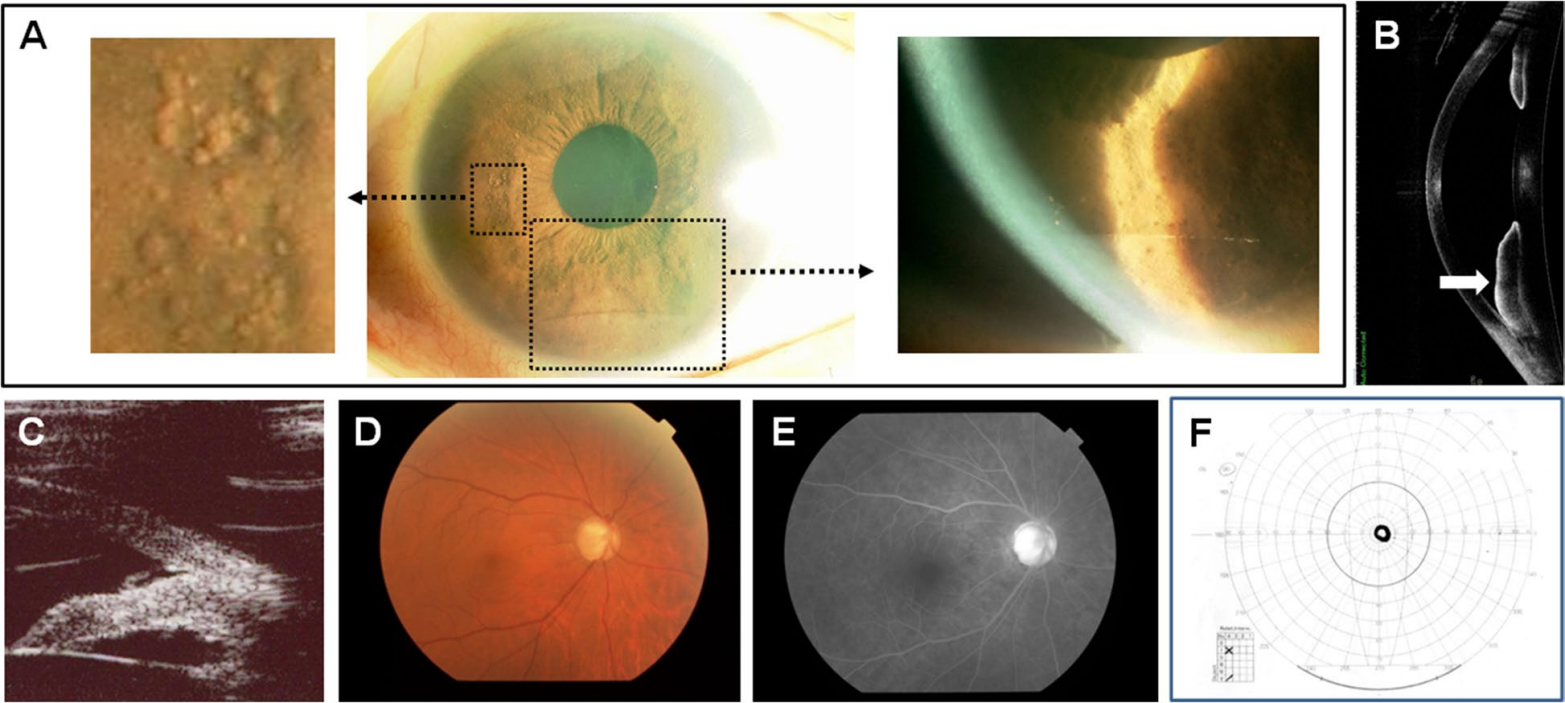
Slit-lamp examination of the anterior ocular segment in the right eye. Numerous iris nodules of different shapes and sizes cover the entire surface of the iris with keratic precipitates. Enlarged views are seen in the separated images (**A**). An AS-OCT image shows thickening of the inferior iris of the right eye (arrow) (**B**). UBM shows angle closure at the 4 o’clock position in the right eye (**C**). A fundus image shows severe glaucomatous optic neuropathy in the right eye (**D**). A late-phase fundus fluorescein angiography image shows hyper-fluorescence of the optic nerve in the right eye (**E**). Goldmann visual field perimetry found that only 5 degrees of the visual field remains functional (**F**)

A 56-year-old woman with iritis and high intraocular pressure (IOP) visited our hospital and reported visual loss in her right eye. The visual acuity (VA) was 20/2000 in the right eye and 20/13 in the left eye. The respective IOPs were 56 mmHg and 18 mmHg measured using Goldmann applanation tonometry. Slit-lamp microscopy revealed a large number of iris nodules of different sizes over the entire surface of the iris with keratic precipitates and mild inflammatory cells in the anterior chamber of the right eye (Fig. 1A). The grade of anterior chamber cells was + 1 based on the Standardization of Uveitis Nomenclature Working group. Peripheral anterior synechiae were seen all around by the gonioscopy. Anterior-segment optical coherence tomography (AS-OCT) (CASIA, Tomey, Nagoya, Japan) showed a thickened inferior iris in the right eye on a B-scan image (Fig. 1B). Ultrasound biomicroscopy (UBM) (Tomey) showed angle closure at the 4 o’clock position in the right eye (Fig. 1C). A late-phase fundus fluorescein angiography image showed hyper-fluorescence of the optic nerve in the right eye characteristic of severe glaucomatous optic neuropathy (Fig. 1D, E). Goldmann visual field perimetry found only central 5 degrees of vision remaining in the right eye (Fig. 1F). In contrast, the Humphrey Field Analyzer (Carl Zeiss Meditec Inc., Dublin, CA, USA) showed that the visual field in the left eye was normal using the 30–2 SITA-Standard program in standard automated perimetry.

**Fig. 2:**
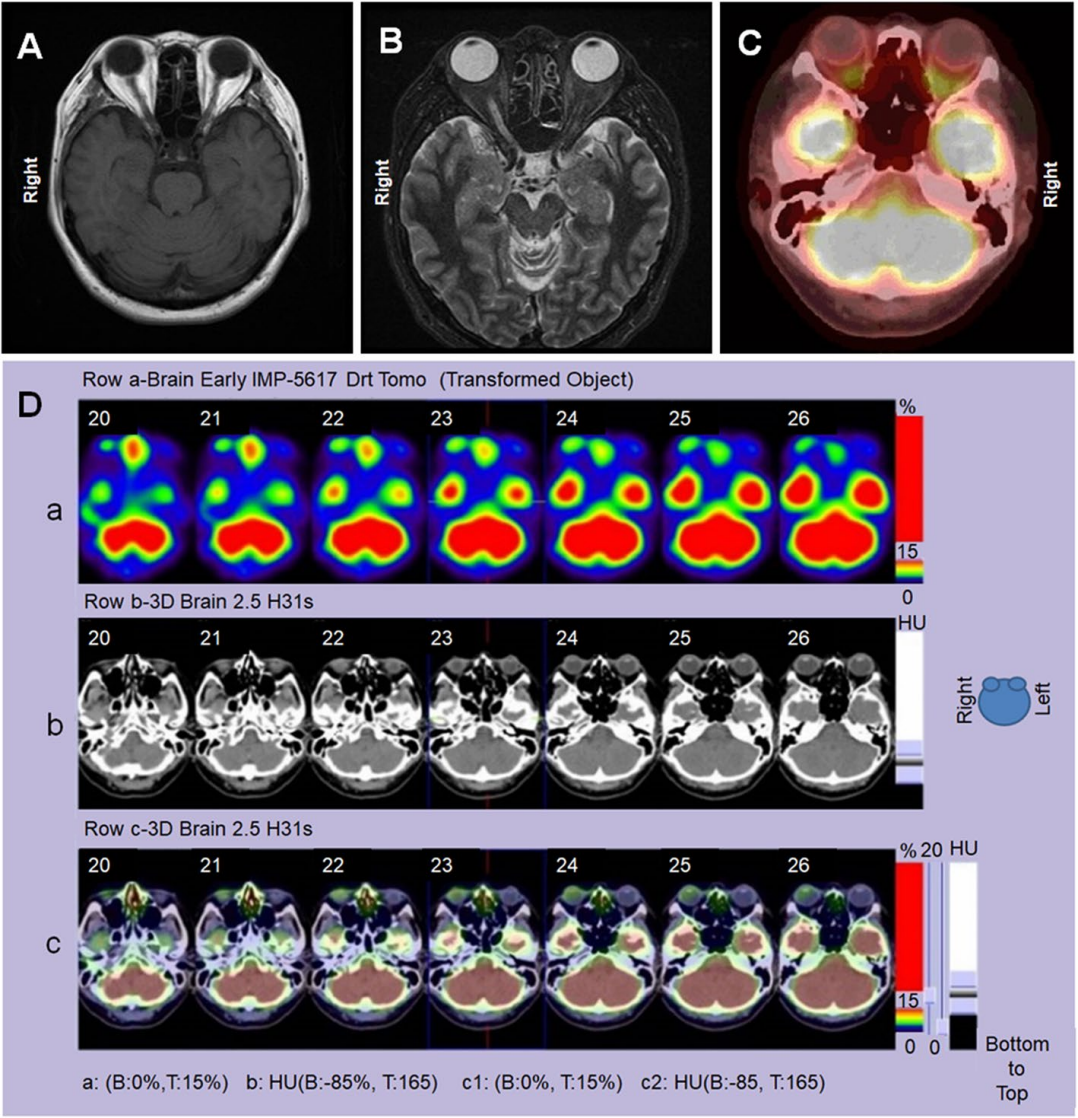
Identification of malignancy by imaging studies. A cranial MRI image shows no abnormality on a T1-weighted image (**A**) and a high signal in the optic nerve of the right eye on a short tau inversion recovery image, but no findings suggestive of malignancy are seen (**B**). A PET-CT also shows no signs of malignancy (**C**). Brain SPECT shows that fluorodeoxyglucose is slightly more concentrated in the right eye than in the left eye in the early images, but no difference is seen between the left and right eyes in the late image and there is no significant accumulation in the trunk (**D**)

The family history was unremarkable. The medical history showed urethral stones and a suspected autoimmune disease 1 year previously, but the details were unknown. Serum testing showed a high rheumatoid factor value (46 IU/mL), high antinuclear antibody titers (1:640), and high Immunoglobulin E levels (226.1 IU/mL). No abnormalities were found on chest radiographs and electrocardiograms. Five-S cysteinyl-dopa (5-S-CD), a melanin-related metabolite, was measured in the serum and aqueous humor to identify a melanin-related tumor such as malignant melanoma [8]. The value was 6 nmol/L in the serum, which was within the normal range (1.5–8.0 nmol/L); however, the value was 9.5 nmol/L in the aqueous humor, which was slightly higher than the normal range [8]. To determine if the tumor was malignant, brain magnetic resonance imaging (MRI) and positron emission tomography-computed tomography (PET-CT) were performed but showed no obvious findings suggestive of a malignancy (Fig. 2A-C). In the brain, an early single photon emission computed tomography (SPECT) image showed slight accumulation of fluorodeoxyglucose in the right eye compared with the left eye, but there was no difference between both eyes in the late images (Fig. 2D). These results suggested the low possibility of a malignancy.

The IOP in the right eye decreased to within the normal range by treatment with a dexamethasone eye drops 4 times daily and multiple anti-glaucoma eye drops, but a few months later the IOP increased to over 40 mmHg and was unresponsive to therapy.

**Fig. 3:**
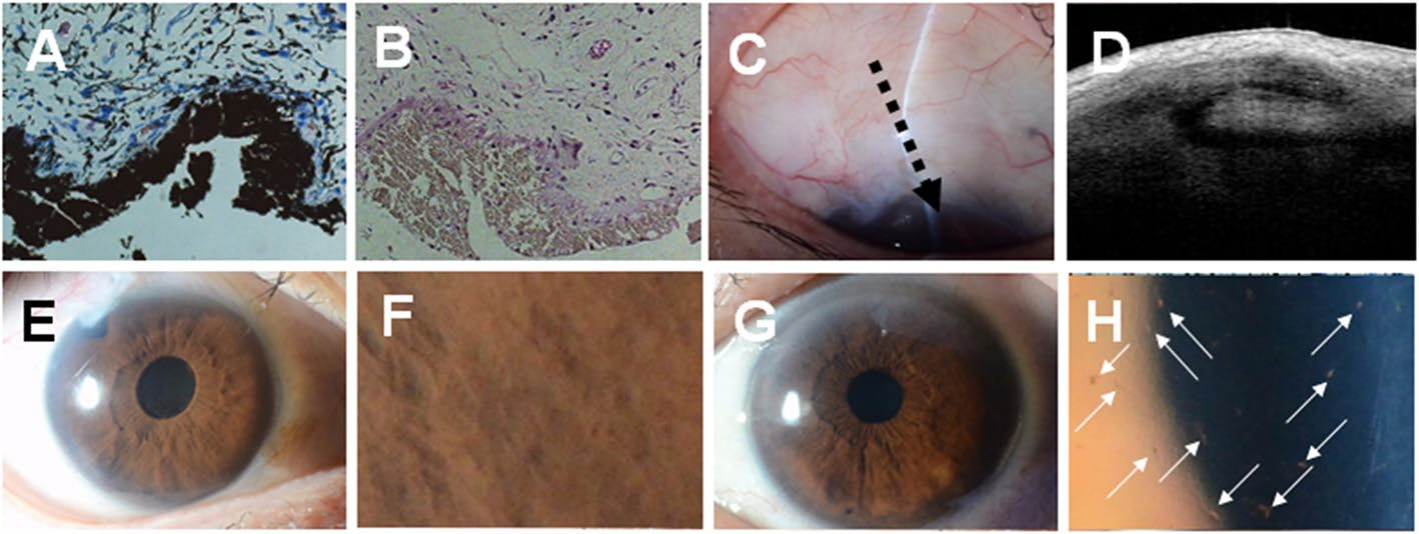
Histology of the intraoperatively resected iris is shown in A and B. A Giemsa stained image shows no obvious papillary structures (**A**). After de-melaninization, pigment epithelial cells show little nuclear atypia and no highly atypical cells causing stromal infiltration. The stroma is edematous and slightly infiltrated by chronic inflammatory cells. No findings suggest malignancy (**B**). A slit-lamp image and morphology of the filtering bleb by AS-OCT 7 years postoperatively are shown in C and D. A photograph of the anterior segment shows a well-maintained filtration bleb (**C**). The filtration bleb contains a large reticular layer and is in good condition in the AS-OCT bleb image (dotted arrow in Fig. C) (**D**). An anterior image shows no hyperemia or obvious inflammation. Posterior iris synechiae are visualized around the pupil (**E**). The multiple iris nodules at disease onset resolved, and the nodules are irregular (**F**). An anterior image of the fellow eye shows no hyperemia or obvious inflammation (**G**), but small linear keratic precipitates are seen (white arrows in Fig. H) (**H**)

We performed a trabeculectomy, and an iris specimen obtained during iridectomy was pathologically diagnosed. Giemsa staining did not show an obvious papillary structure (Fig. 3A). Furthermore, the pigment epithelial cells showed little nuclear dysplasia, and no cells with a high degree of atypia causing stromal infiltration were seen by de-melaninization treatment (Fig. 3B). Slight chronic inflammatory infiltration was found in the interstitium, but there were no positive findings of malignancy. Seven years postoperatively, the inflammation has not recurred. Slit-lamp and AS-OCT evaluations showed that the filtering bleb was maintained, and the IOP was stable at around 10 mmHg with timolol eye drops (Fig. 3C, D). The numerous iris nodules of different sizes seen at the first visit gradually shrank and had a sand-like appearance and were inconspicuous after trabeculectomy; posterior iris synechiae were observed around the pupil (Fig. 3E, F). It was difficult to maintain the VA and residual visual field because of the severe degree of preoperative glaucomatous optic neuropathy. In contrast, small linear keratic precipitates were seen in the left eye; however, no obvious inflammation or increased IOP occurred during the entire course (Fig. 3G, H).

## Discussion and conclusion

We experienced a case of advanced visual field loss with secondary glaucoma associated with multiple iris nodules and iritis. Based on previous reports, this case was diagnosed as iris mammillation. The disease generally is unilateral, but in the current case mild findings appeared in the opposite eye over time. The IOP in the right eye decreased to within the normal range by treatment with a dexamethasone eye drops for inflammation and multiple anti-glaucoma eye drops, but finally, the IOP increased again and was unresponsive to therapy because of peripheral anterior synechiae. The patient underwent filtration surgery after a malignancy, such as melanosis, was ruled out because of the potential for metastasis resulting from surgical intervention. Melanin is a characteristic of malignant melanoma, and 5-S-CD most sensitively reflects the clinical pathology of melanoma, leading to early detection and recurrence of malignant melanoma. Measurement of 5-S-CD is useful for determining the therapeutic effect as an index for estimating metastasis. Normally, 5-S-CD is determined by quantifying the amount in the serum, but quantification in the anterior chamber is useful [8]. The serum 5-S-CD level in this case was 6 mol/L, which was within the normal range, but it was 9.5 nmol/L in the aqueous humor, which was slightly above the normal range. Furthermore, PET-CT and brain SPECT were performed to rule out findings suggestive of obvious malignancy. Filtration surgery was performed without lensectomy to minimize surgical invasiveness. An iris specimen excised by iris peripheral iridectomy was treated with de-melaninization as a pathological tissue, and no obvious malignancy was identified. While, inflammatory cell infiltration was observed　in the resected iris, which may have contributed to the series of elevated IOP. Furthermore, multiple nodules on the iris surface gradually shrank and were inconspicuous after trabeculectomy, suggesting that uveitic glaucoma is associated with iris mammillation. The filtering bleb was well maintained with low IOP for 7 years postoperatively, but the VA eventually deteriorated because less than the central 5 degrees of the visual field remained at the first visit. In addition, there was no recurrence of inflammation, and no obvious malignant findings were observed locally or systemically, but careful follow-up is required for the dominant left eye [9]. Iris mammillation is generally unilateral, but a bilateral case was reported [10].

We experienced a case of multiple iris nodules that developed inflammatory secondary glaucoma and were able to control the IOP for a long period after filtration surgery. A preoperative examination to rule out a malignancy is important both locally and systemically when performing filtration surgery with neoplastic lesions. Furthermore, careful follow-up is necessary during a long postoperative period.

## Data Availability

All data generated or analyzed during this study are included in this published article.

## Abbreviations

IOP: Intraocular pressure
VA: Visual acuity
AS-OCT: Anterior-segment optical coherence tomography
MRI: Magnetic resonance imaging
5-S-CD: Five-S cysteinyl-dopa
PET-CT: Positron emission tomography-computed tomography
SPECT: Single photon emission computed tomography
